# Distribution of Node Characteristics in Evolving Tripartite Network

**DOI:** 10.3390/e22030263

**Published:** 2020-02-25

**Authors:** Ladislav Beranek, Radim Remes

**Affiliations:** Department of Applied Mathematics and Informatics, Faculty of Economics, University of South Bohemia, 37005 Ceske Budejovice, Czech Republic; inrem@ef.jcu.cz

**Keywords:** complex systems, tripartite network, evolving network, strength distribution

## Abstract

Many real-world networks have a natural tripartite structure. Investigating the structure and the behavior of actors in these networks is useful to gain a deeper understanding of their behavior and dynamics. In our paper, we describe an evolving tripartite network using a network model with preferential growth mechanisms and different rules for changing the strength of nodes and the weights of edges. We analyze the characteristics of the strength distribution and behavior of selected nodes and selected actors in this tripartite network. The distributions of these analyzed characteristics follow the power-law under different modeled conditions. Performed simulations have confirmed all these results. Despite its simplicity, the model expresses well the basic properties of the modeled network. It can provide further insights into the behavior of systems with more complex behaviors, such as the multi-actor e-commerce system that we have used as a real basis for the validation of our model.

## 1. Introduction

Complex network structures can be detected in many systems that can be found in the field like biology, ecology, social sciences, or large information infrastructures. Many empirical findings have uncovered the general occurrence of complex topological structures underlying many of these networks. For example, online systems based on Internet technology have mostly complex arrangement and different relationships between the entities, which affects their properties.

These topological functions of networks prove to be extremely important as they have a strong impact on the characteristics of networks such as robustness or vulnerability [1,2] and others. In various contexts, the development of a complex network topology was considered as an output of a dynamic system with state variables associated with edges and nodes; see [3,4,5]. The dynamics of these actors (nodes and edges) and their connection patterns influence evolution of networks, their structure and processes ongoing on affected networks. However, the macroscopic behavior of these complex systems can often be reproduced using a suitable network model, with very few assumptions about the components themselves, as also shown herein.

### 1.1. Related Works

Various network models have been formulated in order to describe the network architecture of these systems and to analyze dynamic processes that take place on their structure [6,7,8,9,10,11,12,13,14,15]. One example of such model is a bipartite network. It is widely applied in the modeling of various online platforms, such as online services where users view or purchase products [16,17,18] and listen to music [19], and also in biology [20,21,22], in medical science [23,24], and in other areas [25,26,27,28,29,30]. For example, Saavedra et al. have introduced two mechanisms, specialization and interaction, that lead to the exponential distribution of degrees for both parts of the bipartite network [31]. They found out that bipartite network can effectively characterize the structure of ecological and organizational networks. Further empirical analyses of some models of bipartite networks describing online services [32,33,34,35] have shown that user-level distributions truly follow a shifted power-law, while the distribution of an object’s level always follows the power law. Zhang et al. [36] suggested an evolutionary model of unweighted online bipartite networks. Their theoretical analysis has not only shown that the model can effectively reproduce two different degree distributions but has demonstrated its results on two real data sets, Delicious and CiteULike. The above studies deal with unweighted bipartite networks. However, weighted networks are more appropriate for modeling of online systems. Therefore, Zhang et al. [37] developed this work and proposed a model to describe distribution characteristics for weighted bipartite evolving networks.

Generally, the network system is defined by patterns of different links between agents or various entities. The research on social networks has greatly advanced in understanding how features such as links, paths, position, and other structural parameters determine the possibilities of a group of agents to influence other agents within the network [33]. For example, taking a central position is generally considered to be beneficial because of the relative simplicity of controlling the information distribution and a possibility of influencing other agents. Likewise, the central position can form an information broker who accesses and integrates information through social links [38,39,40].

### 1.2. Contribution

In this paper, we deal with a situation where intermediaries enter relationships modeled initially by a bipartite network and take a central position between the two parts of this formerly bipartite network. They affect relationships and change the network topology into a tripartite network. Their aim is to take the most significant position within the network and to have benefits from this position [38]. In our paper, we study how this addition of intermediaries will affect the properties of this tripartite network. We not only consider the increasing mechanism in the corresponding sets of nodes, but we also take into consideration the decline in strength of some objects and intermediaries. Then, we study the properties of the studied network, changes in strength of objects and intermediaries over time, and their distribution using the mean field method according to [41]. We performed simulations that confirmed the results of our analysis.

Detailed studies of the structural properties and dynamics of tripartite networks are not widely found in the literature. Most papers are concerned with the problem of a detection of communities in tripartite networks [42,43]. Our work is one of the first to discuss the characteristics and evolution of the tripartite network whereby it builds on the past research on bipartite networks [36,37].

**Figure 1:**
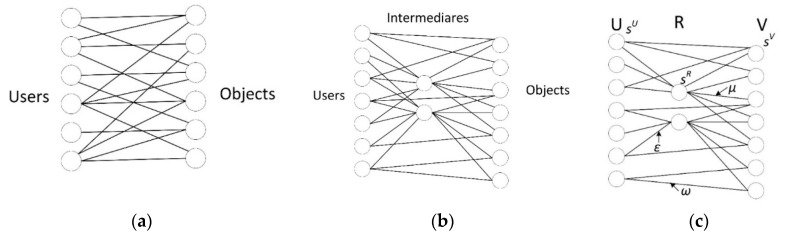
(**a**) Bipartite network, (**b**) tripartite network (our model, e.g., network with intermediaries), and (**c**) marking of edge weights and node strengths of individual actors.

Our research is motivated in real life, in an online business environment. Users can purchase some product directly in a specific e-shop (corresponding model is a bipartite network, see Figure 1a) or they can use an intermediary (price comparison website) to get through this website to some e-shops where they can purchase products (see Figure 1b). A tripartite network can represent relationships in this environment, and the dynamic aspects of this network can be explored. The tripartite network model could be used to study a situation where a traditional political structure with two main parties is questioned by the presence of a new candidate; a detailed analysis of the problem can be found, e.g., in [44].

### 1.3. Paper Structure

Next paper chapters are structured as follows. Section 2 describes in detail our network model and corresponding mechanisms. In Section 3, we present our experiments, the results of analyses and simulations. Section 4 follows and contains a discussion of the results. Conclusions are introduced in Section 5.

## 2. The Model

In this section, we propose a model to find out characteristics of a network originally bipartite after inserting new nodes (and edges) between both parties (users and objects) of the original network (see Figure 1a,b). Our model is based on three associated dynamical mechanisms: topological growth, weight dynamics, and nodes strength dynamics:

**(i) Growth**. Initially, we assume only relationships among users and objects (Figure 1a). Their relationship can be described using a bipartite graph *G* (*U*, *V and E*). We assume the existence of initial sets of nodes *U*_0_ and *V*_0_ and edges *E*_0_. Beginning with these nodes’ seeds, new nodes are being added:

1. At the beginning, a node of a new type, the intermediary r∈R (*R* denotes the set of these entities) enters the existing relations between *U* and *V*. The original bipartite network becomes tripartite one with edges between sets *U* and *V*, *U* and *R*, and *R* and *V* (see Figure 1b,c).

2. In the next time steps, new nodes $v_{i}\in V$, $r_{i}\in R$, or $u_{i}\in U$ are gradually added to the evolving tripartite network. Each new node will be connected to *n* already existing nodes, with high strength nodes being preferred; i.e., we assume preferential connections [6].

**(ii) Dynamics of weights**. The weight of each new edge is initially set to *ω*_0_, *ε*_0_, *μ*_0_ (see Figure 1c). Creating this new edge represents changes in network traffic. For the sake of simplicity, we limit ourselves to the case where the introduction of a new edge on node *i* will trigger only local rearrangements of weights (see Appendix A, Figure 1A). The weights between nodes *i* and *j* in the time step *t* is denoted by *ω_ij_*(*t*) (or *ε*_ij,_ *μ*_ij_). We suppose that *ω_ij_*(*t*) = *ω_ji_*(*t*) (or *ε*_ij_ = *ε*_ji_ , *μ*_ij_ = *μ*_ji_), see Figure 1c.

The edges mean the interaction between users and objects (or intermediaries). The weights of edges indicate the amount of usage between users and objects (or intermediaries) in the sets U, V, and R of their respective nodes. We consider not only the mechanism for increasing the weights of the edges in the two relevant sets of nodes, but also the possibility of edges removing if the intermediary service is refused. Initial values of weights may correspond in real case of e-commerce, for example, to the average value of transactions. In our case, these values correspond to the ratio of the number of accomplished transactions to the number of visited objects (e-shops or intermediaries) (see Appendix C).

**(iii) Node dynamics**. In this paper, we use the node strength that is a generalization of a node degree [45]. Node dynamics is based on the following mechanisms that affect node strength: The strength of some nodes may decrease, or some edges may disappear (e.g., an object *v* rejects connection to an intermediary *r*). The strength of node i∈V (new object) that is added in the time step *t* is denoted as $s_{i}^{V}\left(t\right)$ and is defined as $s_{i}^{V}\left(t\right)=\;\sum_{i\in \Upsilon \left(i\right)}\omega_{ij}$, where Υ(i) denotes a set of the new neighbors of node *i* [45,46] (also the number of new edges). The initial strength of this node is $s_{i}^{V}\left(0\right)=k_{i}\omega_{i}$, where *k*_i_ is the number of the neighbors of node *i*. The same is true for the strength $s_{i}^{U}\left(t\right)$ of nodes i∈U (users) and the strength $s_{i}^{R}\left(t\right)$ of nodes i∈R (intermediaries).

The strength of a node combines the information about its connectivity and the intensity of the weights of its edges. In the case of electronic business, the strength *s_i_* of the node *i* corresponds to the size of the achieved transactions with this node. Another example might be airports network where the strength of the node *i* corresponds to the total traffic passing through this node, indicating the importance of the airport *i*. In the case of a scientific collaboration network, strength indicates the number of articles created by a chosen scientist including IF, see, e.g., [47].

However, it should be emphasized that the correlations between mass and topological properties are encoded in statistical relationships between these quantities. In fact, *s_i_*, which is the sum over all neighbors *i*, is correlated with its degree *k_i_*. In the simplest case of random, uncorrelated masses *w_ij_* with an average 〈*w*〉, the strength is *s* ~ 〈*w*〉*k*. In the presence of a correlation between weights and topology, it is possible to observe more complex behavior with *s* ~ *Ak*^β^ with *β* ≠ 1 or with *β* = 1 and A ≠ 〈*w*〉 [48]. We use the term “strength of nodes” rather than “degree of nodes” here. The aim is to give an idea of possible dynamics of nodes and edges that can exist in various context. However, in this paper we focus rather on the mechanisms that drive the development of the entire network. In the following sections, we describe these network evolution mechanisms in detail:

### 2.1. Growth of the Network

#### 2.1.1. The Addition of a New User

A new user is added in the time *t* with the probability *p*_1_, and the weights are reset locally [37,45]. We assume that this new user establishes a relationship only with an intermediary in this phase. This new user selects an intermediary based on preferential connection (*n*_1_ edges are created among this new user and intermediaries):

(1) $$\Pi \left(s_{j}^{R}\right)=\frac{s_{j}^{R}\left(t\right)}{\sum_{i\in R}s_{i}^{R}\left(t\right)}$$

**Figure A1:**
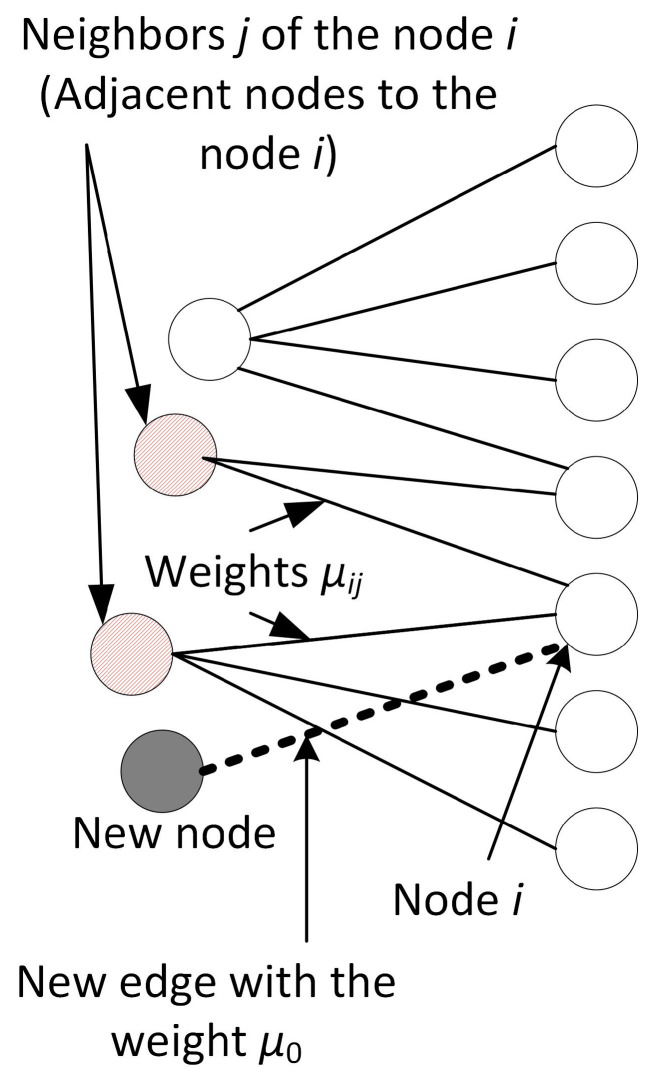
Illustration of changes in weight of existing edges after addition of a new edge.

We assign an initial strength $s_{j}^{U}\left(0\right)$ to the new user. If this new object node connects to the intermediary (node) *j*, it triggers variations of the existing weights across the network. We focus on the case where the addition of a new edge with weight *ε*_0_ induces the local rearrangements [37,45] of weight between *j* and its other neighbors i∈Υ(j) according to the rule (see Figure A1 in Appendix A):

(2) $$\varepsilon_{ij}\left(t\right)=\varepsilon_{0}+\delta_{1}\frac{\varepsilon_{ij}\left(t-1\right)}{s_{j}^{R}\left(t-1\right)},$$

where *ε*_0_ is an initial weight of the new edge and *δ*_1_ is a parameter of increasing the weight of relevant existing edges influenced by creation of a new edge from a new user to an intermediary, see also Appendix C.

#### 2.1.2. The Addition of a New Object

A new object is added in the time *t* with the probability *p*_2_, and the weights are reset locally [37,45]. We also assume here that a new object establishes a relationship only with an intermediary in this initial phase (we do not assume the creation of edges between this object and users at this stage). This new object selects an intermediary based on preferential connection (*n*_2_ edges are created among new object and intermediaries):

(3) $$\Pi \left(s_{j}^{R}\right)=\frac{s_{j}^{R}\left(t\right)}{\sum_{i\in R}s_{i}^{R}\left(t\right)}.$$

We assign an initial strength $s_{j}^{V}\left(0\right)$ to the new object. If this new object connects to the intermediary *j*, it triggers variations of the existing weights across the network. We focus on the case where the addition of a new edge with weight *µ*_0_ induces the local rearrangements [37,45] of weight between *i* and its other neighbors i∈Υ(j) according to the rule (see Figure A1 in Appendix A):

(4) $$\mu_{ij}\left(t\right)=\mu_{0}+\delta_{2}\frac{\mu_{ij}\left(t-1\right)}{s_{j}^{R}\left(t-1\right)},$$

where *μ*_0_ is an initial weight of the new edge and *δ*_2_ is a parameter.

#### 2.1.3. The Addition of a New Intermediary

A new intermediary is added in the time step *t* with the probability *p*_3_. The initial strength $s_{i}^{r}\left(0\right)$ is assigned to it. This intermediary will form connections to objects based on the preferential connection (*n*_3_ edges are created):

(5) $$\Pi \left(s_{j}^{O}\right)=\frac{s_{j}^{O}\left(t\right)}{\sum_{i\in R}s_{i}^{O}\left(t\right)}.$$

This transaction will affect the weight of the edges through the relationship (a new edge with weight *µ*_0_ induces the local rearrangements of weight between *i* and its other neighbors i∈Υ(j)):

(6) $$\mu_{ij}\left(t\right)=\mu_{0}+\delta_{3}\frac{\mu_{ij}\left(t-1\right)}{s_{j}^{V}\left(t-1\right)}.$$

### 2.2. The Changes of Edges’ Weight

At the time step *t*, a certain number of already existing users selected based on the preferential connection (Equation (7))

(7) $$\Pi \left(s_{j}^{U}\right)=\frac{s_{j}^{U}\left(t\right)}{\sum_{i\in U}s_{i}^{U}\left(t\right)}$$

that will connect with nodes already existing in *V* (objects) or in *R* (intermediaries) using one of the following mechanisms:

(i) with *n*_4_ nodes already existing in *V* (objects) and with the probability *p*_4_ (*n*_4_ edges are created) based on the preferential attachment:

(8) $$\Pi \left(s_{j}^{V}\right)=\frac{s_{j}^{V}\left(t\right)}{\sum_{i\in V}s_{i}^{V}\left(t\right)}.$$

This transaction will affect the weight *ω*_ij_ of the edge between the user and objects; we can express this change as follows:

(9) $$\omega_{ij}\left(t\right)=\omega_{0}+\omega_{ij}\left(t-1\right)+\delta_{4}\frac{\omega_{ij}\left(t-1\right)}{s_{j}^{V}\left(t-1\right)}+\delta_{4}\frac{\omega_{ij}\left(t-1\right)}{s_{i}^{U}\left(t-1\right)},$$

where *ω*_0_ is an initial weight of the new edge.

(ii) users choose an intermediary with the probability *p*_5_ based on the preferential connection (*n*_5_ edges are created) according to Equation (1). This transaction will affect the edge weight between a user and an intermediary through the relationship:

(10) $$\varepsilon_{ij}\left(t\right)=\varepsilon_{0}+\delta_{5}\frac{\varepsilon_{ij}\left(t-1\right)}{s_{j}^{R}\left(t-1\right)}+\delta_{5}\frac{\varepsilon_{ij}\left(t-1\right)}{s_{i}^{U}\left(t-1\right)}.$$

where *ε*_0_ is the initial weight of the new edge.

At the same time, selected intermediaries will make connection with *m* existing nodes in *V* based on the preferential connection according to Equation (8). This transaction will affect the weight of the edges that we express by the equation (we only consider the local shifts of the weight):

(11) $$\mu_{ij}\left(t\right)=\mu_{0}+\delta \frac{\mu_{ij}\left(t-1\right)}{s_{j}^{V}\left(t-1\right)}+\delta \frac{\mu_{ij}\left(t-1\right)}{s_{i}^{R}\left(t-1\right)}$$

### 2.3. The Changes of the Strength of Nodes

#### 2.3.1. The Decreasing of the Strength of an Object

At a certain time, the strength of object *i* will decrease with the probability *p*_6_. The relevant object *i* will be selected by the anti-preferential probability:

(12) $$P\left(s_{i}^{V}\right)=\frac{1-\;\frac{s_{i}^{V}\left(t\right)}{\sum_{j\in V}s_{j}^{V}\left(t\right)}}{\mu_{0}+t\cdot p_{2}-1}$$

The decrease of the strength of node *i* can be formulated [37,45]:

(13) $$s_{i}^{V}\left(t\right)=\left(1-\gamma_{1}\right)s_{i}^{V}\left(t-1\right).$$

#### 2.3.2. The Refusal of Intermediary Service

We suppose that large objects can reject with the probability *p*_7_ connection to an intermediary *j* (see example of the real situation in [49]). The probability that refusal will be realized by the node *i* with the greatest strength $s_{i}^{V}$ can be expressed by Equation (5). The strength of this object *i* is reduced by the weight value of the edge$\;\mu_{ij}$ that disappears:

(14) $$s_{i}^{V}\left(t\right)=s_{i}^{V}\left(t-1\right)-\mu_{ij}\left(t-1\right)$$

This refusal will have an impact on intermediary whose service the object *i* has used. At the same time, the strength of this intermediary will be reduced by the weight of the respective edge to the intermediary *j* that no longer exists. This reduction also affects the surroundings of the intermediary *j*, thus reducing the weight of the node edges *k*, for which k∈Υ(j), by the relation:

(15) $$s_{j}^{R}\left(t\right)=s_{j}^{R}\left(t-1\right)-\mu_{ij}\left(t-1\right)-\underset{k\in \Upsilon \left(j\right)}{\sum }\delta \frac{\mu_{kj}(t-1)}{s_{k}^{V}\left(t-1\right)}$$

The decreasing of the strength of an intermediary *j* with the probability *p*_8_ with the anti-preferential probability:

(16) $$P\left(s_{i}^{R}\right)=\frac{1-\;\frac{s_{i}^{R}\left(t\right)}{\sum_{j\in R}s_{j}^{R}\left(t\right)}}{\mu_{0}+t\cdot p_{3}-1}$$

If we select node *i*, then the weight of the edge *µ_ij_* is reduced for all nodes *j* with which *i* has a common edge, i.e., for j∈Υ(i), by the relation:

(17) $$s_{i}^{R}\left(t\right)=\left(1-\gamma_{2}\right)s_{i}^{R}\left(t-1\right).$$

## 3. Simulations and Results

At each time step, one and only one of these described mechanisms is at work, and which one is at work is controlled by its probability parameter *p_i_*. These probability parameters are the regulatory parameters that control the increasing number of objects, users, and intermediaries. The parameters *n_i_* are the control parameters that control the addition of edges. Other parameters *δ_i_* and *r_i_* are the control parameters that control the increase or decrease of edge weight.

Topology properties of this evolving network can be derived from the strength distributions. The node strengths varying at the time *t* are intermediate variables to obtain strength probability distributions for objects and intermediaries. In the performed simulations, we investigated the effect of the probability of the choice of an object directly or using intermediary on distribution characteristics of objects and intermediaries. We also analyzed changes of these characteristics in a situation when the strength of some intermediaries was decreasing, or when some objects decided not to refuse the connections to (services of) intermediaries. The results of the analytical solution of these strength distributions are described in the next sections together with simulation results. The procedure for obtaining data for our model validation and their values used in simulations are given in Appendix C and Table A1.

### 3.1. Analytical Solution

The analytical solution of the object’s strength distribution is described in detail in Appendix A. The change of the strength of objects over time *t* is given by the equation:

(18) $$s_{j}^{V}\left(t\right)\approx n_{2}\mu_{0}{\left(\frac{t}{t_{j}}\right)}^{\Gamma_{3}}$$

The strength distribution $P\left(s^{V}\right)$ of objects in dependence on strength $s^{V}$:

(19) $$P\left(s^{V}\right)=H{\left(s^{V}\right)}^{-\xi }$$

where $\Gamma_{3}$, *H*, and *ξ* are formulated by Equations (A15), (A20) and (A21), respectively.

The analytical solution of the strength distribution of intermediaries is described in detail in Appendix B. The change of the strength over time *t* is given by the equation:

(20) $$s_{j}^{R}\left(t\right)\approx n_{3}\mu_{0}{\left(\frac{t}{t_{j}}\right)}^{\Gamma_{3}^{’}}$$

The strength distribution $P\left(s^{R}\right)$ of intermediaries in dependence on strength $s^{R}$:

(21) $$P\left(s^{R}\right)=H’{\left(s^{R}\right)}^{-\xi^{’}}$$

where $\Gamma_{3}^{’}$, resp. *H*’, and resp. *ξ*’ are formulated by Equation (A30), resp. (A33), and resp. (A34), respectively.

The scaling exponents $\Gamma_{3}$ and $\Gamma_{3}^{’}$ of strength distribution as a function of parameters have been derived by using the rate equation and mean-field method [41] (see Appendix A and Appendix B). The results of analytical solution show that the strength distributions of intermediaries and of objects in our tripartite network follow the power-law distribution.

### 3.2. Distribution Characteristics and Users’ Choice of Connection to Objects

**Figure 2:**
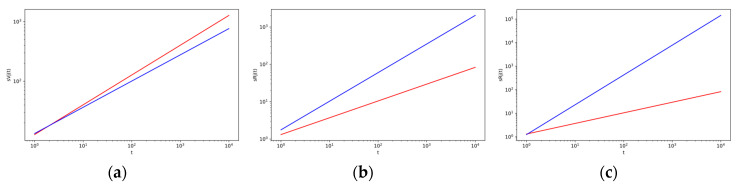
The log–log plot of the change of strength $s_{j}^{V}\left(t\right)$ (**a**, on the left) and the log–log plot of the change of strength $s_{j}^{R}\left(t\right)$ (**b**, in the middle, and **c**, on the right) with the time *t* for different values of *p*_4_ and *p*_5_ and with other fixed parameters given in Table A1 in Appendix C, with *t_j_* = 2. The lines correspond to the theoretical results, red line in Figure 2a,b is for the values *p*_4_ = 0.630, *p*_5_ = 0.309, blue line is for the values *p*_4_ = 0.309, *p*_5_ = 0.630, and the corresponding marks for the simulation results are red (∗) and blue (△). In (**c**), the results are shown for the values *p*_3_ = 0.630, *p*_4_ = 0.309 (red line), and *p*_4_ = 0.05, *p*_5_ = 0.88 (blue line).

**Figure 3:**
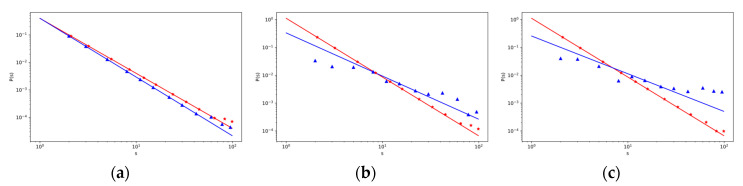
The log–log plot of objects strength distribution $P\left(s^{V}\right)$ changes over objects strength $s^{V}$ (**a**, left) and the log–log plot of intermediary strength distribution $P\left(s^{R}\right)$ changes over intermediary strength $s^{R}$ (**b**, in the middle, and **c**, on the right) for different values of *p*_4_ and *p*_5_ with other fixed parameters given in the Table A1 in Appendix C. The lines correspond to the theoretical results, red line in (**a**,**b**) is for the values *p*_4_ = 0.630 and *p*_5_ = 0.309, respectively, and blue line is for the values *p*_4_ = 0.309 and *p*_5_ = 0.630, and the corresponding marks for the simulation results are red (∗) and blue (△). In (**c**), the results are shown for the values *p*_4_ = 0.309 and *p*_5_ = 0.630, blue line is for the values *p*_4_ = 0.05 and *p*_5_ = 0.88, and the corresponding marks for the simulation results are red (∗) and blue (△). The comparison between simulation and analytic results for weight distributions with system sizes t = 4000.

Figure 2 and Figure 3 show the example of the effect of the way the users use to come to objects on the strength characteristics of chosen objects and intermediaries. Figure 2a shows how the strength of an object that was added at time *t_j_* = 2 changes over time. Figure 2b,c shows similarly how the strength of an intermediary, which was also added at time *t_j_* = 2, changes over time. A blue line indicates a greater probability that users will come to the objects through an intermediary.

Although the probability values *p*_4_ and *p*_5_ are quite different in these cases, the impact of the way users come to some objects on the strength of the object and its distribution function is not very significant (under the other fixed values of parameters given in Table A1 in Appendix C, and various *p*_4_ and *p*_5_). This fact is confirmed in Figure 2a and Figure 3a.

Figure 3b,c show the distribution characteristics of intermediaries for various *p*_4_ and *p*_5_. Intermediaries added to the system later also have a chance of gaining more strength under given model conditions. The number of intermediaries with great strength is increasing with greater probability *p*_5_ as shown in Figure 3c. Great intermediaries can gain a key position throughout the analyzed network.

This result and the course of the distribution characteristics was confirmed by our simulation calculations (red (∗) mark lower *p*_5_ and blue (△) mark for higher *p*_5_).

### 3.3. The Effect of Canceling a Connection to Some Intermediary

**Figure 4:**
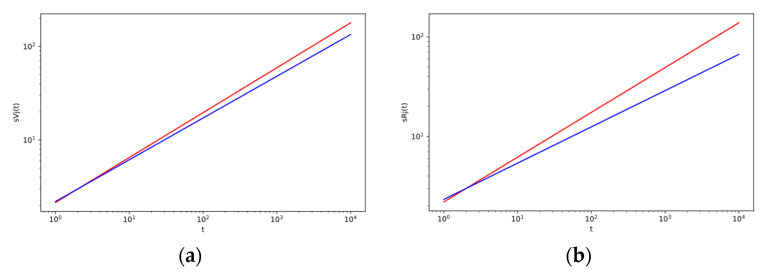
The log–log plot of change of strength $s_{j}^{V}\left(t\right)$ (**a**, left) and the log–log plot of the change of strength $s_{j}^{R}\left(t\right)$ (**b**, on the right) with the time *t* for different values of *p*_7_ (probability that the object will refuse to use the service of an intermediary) with other fixed parameters given in Table A1 in Appendix C, with *t_j_* = 2. Red line is for value *p*_7_ = 0, and blue line is for value *p*_7_ = 0.02.

**Figure 5:**
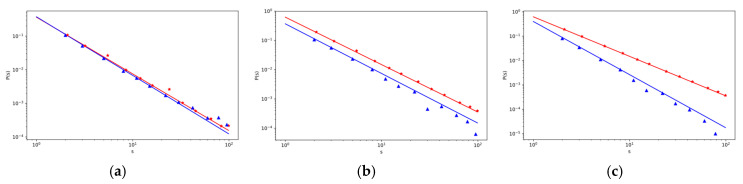
The log–log plot of objects strength distribution $P\left(s^{V}\right)$ changes over objects strength $s^{V}$ (**a**, left) and the log–log plot of intermediary strength distribution $P\left(s_{j}^{R}\right)$ changes over intermediary strength $s_{j}^{R}$ (**b**, in the middle, and **c** on the right) for different values *p*_7_ (probability that the object will refuse to use the service of an intermediary) with other fixed parameters given in the Table A1 in Appendix C. The lines correspond to the theoretical results, red line is for value *p*_7_ = 0, blue line is for value *p*_7_ = 0.01 in (**a**,**b**), and the corresponding marks for simulation results are red (∗) and blue (△). In (**c**), the results are shown for the values *p*_7_ = 0 (red line) and *p*_7_ = 0.05 (blue line), and the corresponding marks for simulation results are red (∗) and blue (△). The comparison between simulation and analytic results for weight distributions with system sizes t = 4000.

Figure 4 and Figure 5 show the example of the effect when an object cancels the connection to an intermediary (they refuse to continue in the use of the service of an intermediary). Users must only access this object directly. In the real situation, large e-shops may refuse to use services of some intermediary (certain real situation is described in [49]). This decision may have a well-founded basis, as shown in Figure 3a. There is no big difference between the distribution function of objects that users access more directly (p4 = 0.630, red line) and the distribution function of objects where users access objects rather through an intermediary (p5 = 0.630, blue line). In the previous section, we mentioned that the intermediator function does not have a major influence on the strength of an object, especially a large one, under given conditions. This is also reflected in Figure 4 and Figure 5. Conversely, this decision of some objects can have a significant effect on the intermediary. Their strength distribution characteristics under higher probability of rejection of connection to intermediary (rejection of its services) are shown in Figure 5b,c. It means that in a real situation, an intermediary must attract all objects, especially the big ones.

These conclusions were also confirmed by our simulation calculations. Here, the impact of the cessation of the use of intermediary services by objects was even more evident, especially for intermediaries with high strength, as it is seen in Figure 5b,c (blue triangles). Big objects attract many users who often use intermediaries to access these big objects. If intermediaries lose large objects, they consequently lose many edges. Their strength will decrease.

### 3.4. Effect of Decreasing the Strength of An Intermediary

**Figure 6:**
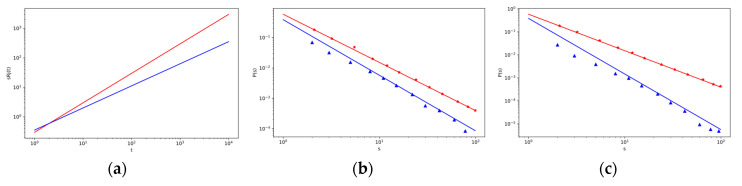
(**a**) The log–log plot of the change of strength $s_{j}^{R}\left(t\right)$ of an intermediary with the time t for different values of *p*_8_ (probability of decreasing the strength of an intermediary) with other fixed parameters given in Table A1 in Appendix C. Red line is for value *p_8_* = 0, and blue line is for value *p*_8_ = 0.01. (**b**,**c**): The log–log plot of intermediary strength distribution $P\left(s^{R}\right)$ changes over intermediary strength $s^{R}$ for different values *p*_8_ (probability of decreasing the strength of an intermediary) with other fixed parameters given in the Table A1 in Appendix C. The lines correspond to the theoretical results, red line is for value *p*_8_ = 0, blue line is for value *p*_8_ = 0.01 in (**b**), and the corresponding marks for simulation results are red (∗) and blue (△). In the (**c**), the results are shown for the values *p*_7_ = 0 (red line) and *p*_7_ = 0.05 (blue line), and the corresponding marks for simulation results are red (∗) and blue (△). The comparison between simulation and analytic results for weight distributions with system sizes t = 4000.

Figure 6a–c show the example of the effect of reducing the strength of intermediaries. The strength of intermediaries with low strength continues to decline. This strength reduction process mainly affects new intermediaries at the beginning when they enter the system, and their strength is still low compared to other intermediaries who are already working in the system. This is evident in Figure 6b and in particular in Figure 6c. The results of the simulations confirm these conclusions. The distribution characteristics of intermediaries are more inclined in the case of a non-zero probability of decreasing intermediaries’ strength. In this case, the low-strength intermediaries even tend to disappear (Figure 6c). The decreasing of the strength of intermediators does not affect distribution characteristics of objects (hence no graphs for objects are shown here).

## 4. Discussion

As part of our work, we conducted a series of simulations and analyses. They demonstrate correlations between structural parameters and the shape of the strength distributions of individual network actors. We found out that the evolving parameters *p*_i_, *γ*_1_, and *γ*_2_ r can be used to control the change in node strength in time step *t*. The exponents of the scaling ξ and ξ’ (or Γ and Γ’) of strength distribution as a function of parameters were derived using the speed equation and middle-field methods. We have shown that the strength distribution of intermediaries and objects is governed by the power law distribution. The results of our analysis and simulation also show that different quantities such as strength, degree, and weights are distributed according to power laws with exponents that are not universal and depend on the specific parameters that control the local microscopic weights’ dynamics. This result is in line with the conclusions set out in [39]. It hints to a simple explanation of the lack of any universality observed in real-world networks.

Despite its simplicity, the model captures the basic characteristics of the modeled real network, the results of the numerical simulations correspond to the theoretical analyses. Furthermore, the performed analyses and simulations showed that the distribution characteristics of objects do not differ greatly under the changing conditions (see previous sections). According to these results (Figure 2a, Figure 3a, Figure 4a), it seems that users and objects can function without an intermediary. Especially at the beginning, intermediaries must, in some way, persuade or compel the users to connect them when accessing the objects. In the real case of Internet platforms, intermediaries (e.g., price comparison sites) may offer some services, for example, it is easier to find information, the mediation of additional services, the mediation of trust, etc. In general, Internet intermediaries (e.g., price comparison sites) are important actors as their services create network externalities. The benefits of using their services increase with the expansion. Therefore, building a critical mass of users is a key factor for them.

Simulations also have shown the key position of intermediaries and indicated the dynamics of their position. For example, Figure 4c shows that as the strength of small intermediaries decreases, the number of intermediaries with great strength increase. Small intermediaries can even disappear. However, this may have the effect that the high-strength objects will not want to be fully dependent on intermediaries [49] and may reject the connection with a high-strength intermediary (Figure 5b,c). It may then experience a phenomenon that is referred to in connection with Internet platforms as disintermediation, the declination in the use of intermediaries and efforts to circumvent them [50].

Our analyses and simulations confirm that the key role of the intermediary consists in the possibility to control interactions in respective networks. However, this control derived from “structural holes” is uncertain and the power of the intermediary only gives him “chances of success” depending on the tensions between relationships [51,52].

A typical application of our model may be a real situation where a new entity (an intermediary) enters central position between the two groups of entities. It enters relationships between two entities (which can be modeled by a bipartite network). This intermediary affects existing relationships and changes the network topology. As aforementioned, the motivation for this paper was the situation where a price comparison website enters into the relationships between customers and e-shops in B2C system [53].

Another example where our model can be used is the modeling of the intermediary’s activity in the electricity market [54] or the Pinterest tripartite network [55]. This network consists of users, boards, pins, and relationships. This network can be analyzed as described in this paper. Credit risk sharing in a multi-stakeholder network (insurance companies, banks, firms) [56] can be also modeled by tripartite network. Described approach can be used in modeling of the emergence of a tripartite structure in a policy created, for example, by two main parties and the presence of a new contender [44] as mentioned in Section Introduction. Many examples of relationships with intermediaries can be found in social relationships [57], ecology [58], citation networks [59], economics [60], and others.

In modeling such a network, data acquisition for model validation is an important problem. For our model, we tried to estimate network parameters that would approximate the real data (see Appendix C). In most cases, data can be elicited with the help of interviews with respondents (to reveal social and economic relations) or various forms of exploration or measurement. In most cases, however, only approximate parameters for modeling can be obtained. Accurate measurement of relationships and their evolution is not usually achievable.

## 5. Conclusions

Complex networks play an essential role in a wide range of disciplines such as social and behavioral sciences, biology, economics, industrial engineering, information technology, and more. By analyzing the structure and behavior of a given network, we can draw useful conclusions when studying various complex systems. In this paper, we use a model of evolving weighted tripartite network with preferential growth mechanisms and different rules for changing the strength of nodes and edge weights. Our goal was to create and to analyze a model of a weighted tripartite network to gain an overview of the role of the single types of nodes (objects and intermediaries). Despite its simplicity, the model captures the essential characteristics of the modeled real network, the distribution of node strength then corresponds to the distribution characteristic of the power law under defined conditions. This weighted tripartite evolving network provides another idea about the behavior of complex systems with more complicated behavior, such as the multi-actor e-commerce system.

In order to create our model more realistic, we are considering further work in the future. First, simulation experiments with different parameter conditions can reveal some more interesting features of modeled complex system based on tripartite network. Secondly, we also want in our future work to refine some parameters expressing the behavior of individual actors in order to model the described system more accurately.

## Appendix A. The Distribution of Strength of Objects

In this section, we derive the distribution of strength of objects. We assume that strength of an *i*-*th* object $s_{i}^{V}\left(t\right)$ is continuous. The corresponding change $s_{i}^{V}\left(t\right)$ in each step is:

A. The addition of a new object, that joins an intermediary (Figure A1), *n*_1_ edges are created with the probability *p*_1_:

(A1) $$\frac{ds_{j}^{V}\left(t\right)}{dt}=p_{2}n_{2}\left(\mu_{0}+\sum_{i\in \Upsilon \left(j\right)}\delta_{2}\frac{\mu_{ij}\left(t\right)}{s_{i}^{R}\left(t\right)}\right)\;\Pi \left(s_{i}^{R}\right)$$

We assume that such an addition of a new object will not affect other objects.

B. The addition of a new intermediary that makes connection with *n*_2_ objects.

(A2) $$\frac{ds_{j}^{V}\left(t\right)}{dt}=p_{3}n_{3}\left(\mu_{0}+\delta_{3}\right)\Pi \left(s_{i}^{V}\right).$$

We assume that the addition of a new intermediary will affect strength of the object that this intermediary join, as well as its surroundings (Figure A1).

C.i. The addition of *n*_3_ edges between existing objects and existing users with the probability *p*_3_:

(A3) $$\frac{ds_{j}^{V}}{dt}=p_{4}n_{4}\left(\omega_{0}+\delta_{4}\right)\Pi \left(s_{j}^{U}\right)+p_{4}n_{4}\underset{j\in \Upsilon \left(i\right)}{\sum }\delta_{4}\frac{\omega_{ij}\left(t\right)}{s_{i}^{R}\left(t\right)}\Pi \left(s_{i}^{V}\right)$$

On the right-hand side of Equation (A3), the first element represents an increase of $s_{i}^{V}\left(t\right)$, in which the change in edge weight is caused only by the node *i* in the time step *t*. The second element denotes an increase of $s_{i}^{V}\left(t\right)$ caused by a change in the weight of the node *j* that is a neighbor of node *i*.

C.ii. The addition of *n*_4_ edges between object and intermediary.

(A4) $$\frac{ds_{j}^{V}}{dt}=p_{5}n_{5}m\left(\mu_{0}+\delta \right)\Pi \left(s_{j}^{R}\right)+p_{5}n_{5}m\underset{j\in \Upsilon \left(i\right)}{\sum }\delta \frac{\mu_{ij}\left(t\right)}{s_{i}^{R}\left(t\right)}\Pi \left(s_{i}^{V}\right)$$

Similar equation but between object and intermediary. On the right-hand side of Equation (A4), the first element represents an increase of $s_{i}^{V}\left(t\right)$, in which the change in edge weight is caused only by the node *i* in the time step *t*. The second element denotes an increase of $s_{i}^{V}\left(t\right)$ caused by the change of the weight of the node *j* that is a neighbor of the node *i*.

D. The decrease the strength of the object.

(A5) $$\frac{ds_{i}^{V}\left(t\right)}{dt}=-p_{6}\gamma_{1}s_{i}^{V}\left(t\right)Ρ\left(s_{j}^{V}\right)$$

E. The rejection of the services of an intermediary by objects.

(A6) $$\frac{ds_{i}^{V}\left(t\right)}{dt}=-n_{7}p_{7}(\mu_{0}+\delta_{3})\Pi \left(s_{i}^{V}\right)$$

In order to get the solution of Equations (A1)–(A7), we will make appropriate adjustments and approximations. In time step *t*, the total strength of nodes belonging to *V* (when neglecting the initial edge weight $\varepsilon_{0}$ and $\omega_{0}$, and for t→∞) is approximately:

(A7) $$\underset{j\in V}{\sum }s_{j}^{V}\left(t\right)\approx p_{2}n_{2}(\mu_{0}+\delta_{2})t+p_{3}n_{3}\left(\mu_{0}+\delta_{3}\right)t+p_{4}n_{4}\left(\omega_{0}+2\delta_{4}\right)t+p_{5}n_{5}m\left(\mu_{0}+2\delta \right)t-p_{7}n_{7}\left(\mu_{0}+\delta_{3}\right)-p_{6}\gamma_{1}\overset{t}{\underset{l=1}{\sum }}s_{l}^{V}\left(l\right)$$

where $s_{l}^{V}\left(l\right)$ is the node strength that goes down in time step *l*. Since the node strength would decrease *r* times at each time step, we can suppose the strength of this node will fall *r* times with probability *p*_5_ when t→∞. Hereby, we can write the previous equation in approximate form:

(A8) $$\underset{j\in V}{\sum }s_{j}^{V}\left(t\right)\approx p_{2}n_{2}(\mu_{0}+\delta_{2})t+p_{3}n_{3}\left(\mu_{0}+\delta_{3}\right)t+p_{4}n_{4}\left(\omega_{0}+2\delta_{4}\right)t+p_{5}n_{5}m\left(\mu_{0}+2\delta \right)t-p_{7}n_{7}\left(\mu_{0}+\delta_{3}\right)-p_{6}\gamma_{1}\underset{j\in V}{\sum }s_{j}^{V}\left(t\right)$$

We simplify this equation and get it in the form:

(A9) $$\underset{j\in V}{\sum }s_{j}^{V}\left(t\right)\approx \frac{p_{2}n_{2}(\mu_{0}+\delta_{2})t+p_{3}n_{3}\left(\mu_{0}+\delta_{3}\right)t+p_{4}n_{4}\left(\omega_{0}+2\delta_{4}\right)t+p_{5}n_{5}m\left(\mu_{0}+2\delta \right)t-p_{7}n_{7}\left(\mu_{0}+\delta_{3}\right)}{1+p_{6}\gamma_{1}}$$

We denote

(A10) $${\;\Gamma }_{1=}\frac{p_{2}n_{2}\left(\mu_{0}+\delta_{2}\right)+p_{3}n_{3}\left(\mu_{0}+\delta_{3}\right)+p_{4}n_{4}\left(\omega_{0}+2\delta_{4}\right)+p_{5}n_{5}\left(\mu_{0}+2\delta \right)-p_{7}n_{7}(\mu_{0}+\delta_{3})}{1+p_{6}\gamma_{1}}$$

where $p_{6}\gamma_{1}>0$.

When t→∞, we can write:

(A11) $$\underset{j\in V}{\sum }s_{j}^{V}\left(t\right)\approx \Gamma_{1}t$$

Now, we combine Equations (A8) to (A12) into one equation:

(A12) $$\frac{ds_{i}^{V}\left(t\right)}{dt}\approx \frac{p_{2}n_{2}\left(\mu_{0}+\delta_{2}\right)s_{i}^{V}\left(t\right)}{\Gamma_{1}t}+\frac{p_{3}n_{3}(\mu_{0}+\delta_{3})s_{i}^{V}\left(t\right)}{\Gamma_{1}t}+\frac{p_{4}n_{4}(\omega_{0}+2\delta_{4})s_{i}^{V}\left(t\right)}{\Gamma_{1}t}+\frac{p_{5}n_{5}m(\mu_{0}+2\delta )s_{i}^{V}\left(t\right)}{\Gamma_{1}t}-\frac{p_{7}n_{7}(\mu_{0}+\delta_{3})s_{i}^{V}\left(t\right)}{\Gamma_{1}t}-\frac{p_{6}\gamma_{1}s_{i}^{V}\left(t\right)}{p_{2}t}+\frac{p_{4}\gamma_{1}s_{i}^{V}\left(t\right)}{p_{2}^{2}t^{2}}+\frac{p_{4}\gamma_{1}s_{i}^{V}\left(t\right)}{p_{2}^{2}t^{2}}$$

Assuming t→∞, equation (A13) can be simplified:

(A13) $$\frac{ds_{i}^{V}\left(t\right)}{dt}\approx \frac{(p_{2}n_{2}(\mu_{0}+\delta_{2})+p_{3}n_{3}(\mu_{0}+\delta_{3})+p_{4}n_{4}(\omega_{0}+2\delta_{4})+p_{5}n_{5}m(\mu_{0}+2\delta )-p_{7}n_{7}\left(\mu_{0}+\delta_{3}\right))s_{i}^{V}\left(t\right)}{\Gamma_{1}t}-\frac{p_{6}\gamma_{1}s_{i}^{V}\left(t\right)}{p_{2}t}$$

The solution of this equation has a form:

(A14) $$s_{i}^{V}\left(t\right)\approx C\cdot t^{\frac{p_{2}n_{2}(\mu_{0}+\delta_{2})+p_{3}n_{3}(\mu_{0}+\delta_{3})+p_{4}n_{4}(\omega_{0}+2\delta_{4})+p_{5}n_{5}m(\mu_{0}+2\delta )-p_{7}n_{7}\left(\mu_{0}+\delta_{3}\right)}{\Gamma_{1}t}-\frac{p_{6}\gamma_{1}}{p_{2}t}}$$

where $\Gamma_{1}>0$.

We can denote the exponent in this equation as:

(A15) $$\Gamma_{3}=\frac{p_{2}n_{2}(\mu_{0}+\delta_{2})+p_{3}n_{3}(\mu_{0}+\delta_{3})+p_{4}n_{4}(\omega_{0}+2\delta_{4})+p_{5}n_{5}m(\mu_{0}+2\delta )-p_{7}n_{7}\left(\mu_{0}+\delta_{3}\right)}{\Gamma_{1}}-\frac{p_{6}\gamma_{1}}{p_{2}}$$

If we assume that we add the node *j* in time *t_j_*, then it holds for its strength $s_{j}^{V}=n_{2}\mu_{0}$ when t → ∞:

(A16) $$s_{j}^{V}\left(t\right)\approx n_{2}\mu_{0}{\left(\frac{t}{t_{j}}\right)}^{\Gamma_{3}}$$

To get the node strength distribution, we use the mean-field method described in detail in [41]. Let P(s) denote the strength distribution that measures the strength probability of a node.

(A17) $$P\left(s_{j}^{V}\left(t\right)<s^{V}\right)\approx P\left(n_{2}\mu_{0}{\left(\frac{t}{t_{j}}\right)}^{\Gamma_{3}}<s\right)=P\left(t_{j}>t{\left(\frac{s^{V}}{n_{2}\mu_{0}}\right)}^{\frac{-1}{\Gamma_{3}}}\right)\;=1-P\left(t_{j}\leq t{\left(\frac{s^{V}}{n_{2}\mu_{0}}\right)}^{\frac{-1}{\Gamma_{3}}}\right)=1-{\left(\frac{s^{V}}{n_{2}\mu_{0}}\right)}^{\frac{-1}{\Gamma_{3}}}$$

(A18) $$P\left(s^{V}\right)=\frac{\partial P(s_{j}^{V}\left(t\right)<s}{\partial s^{V}}\approx \frac{1}{\Gamma_{3}}{\left(n_{2}\mu_{0}\right)}^{\frac{1}{\Gamma_{3}}}s^{\frac{-1}{\Gamma_{3}}-1}$$

Thus, it is possible to write

(A19) $$P\left(s^{V}\right)=H{\left(s^{V}\right)}^{-\xi }$$

where

(A20) $$H=\frac{1}{\Gamma_{3}}{\left(n_{2}\mu_{0}\right)}^{\frac{1}{\Gamma_{3}}}$$

(A21) $$\xi =\frac{1}{\Gamma_{3}}+1$$

## Appendix B. The Distribution of Strength of Intermediaries

In this section, we derive the distribution of strength of intermediaries. We assume that the strength of *i*-th intermediary $s_{i}^{R}\left(t\right)$ is continuous. The corresponding change in $s_{i}^{R}\left(t\right)$ for each mechanism is:

A. The addition of a new user that join intermediaries, *n*_1_ new edges are created:

(A22) $$\frac{ds_{j}^{R}\left(t\right)}{dt}=p_{1}n_{1}\left(\varepsilon_{0}+\delta_{1}\right)\Pi \left(s_{i}^{R}\right)$$

B. The addition of a new object that join intermediaries, *n*_2_ new edges are created:

(A23) $$\frac{ds_{j}^{R}\left(t\right)}{dt}=p_{2}n_{2}\left(\mu_{0}+\delta_{2}\right)\Pi \left(s_{i}^{R}\right).$$

C. The addition of a new intermediary:

(A24) $$\frac{ds_{j}^{R}\left(t\right)}{dt}=p_{3}n_{3}\left(\mu_{0}+\underset{i\in \Upsilon \left(j\right)}{\sum }\delta_{3}\frac{\mu_{ij}\left(t\right)}{s_{i}^{V}\left(t\right)}\right)\;\Pi \left(s_{i}^{V}\right)$$

D.i. The addition of *n*_4_ new edges between existing users and existing intermediaries with the probability *p*_4_.

(A25) $$\frac{ds_{j}^{R}}{dt}=p_{4}n_{4}\left(\varepsilon_{0}+\delta_{4}\right)\Pi \left(s_{j}^{U}\right)+p_{4}n_{4}\underset{j\in \Upsilon \left(i\right)}{\sum }\delta_{4}\frac{\varepsilon_{ij}\left(t\right)}{s_{i}^{R}\left(t\right)}\Pi \left(s_{i}^{R}\right)$$

On the right side of the Equation (A25), the first element represents an increase of $s_{i}^{R}\left(t\right)$, in which the change in the weight of the edge is caused only by the node *i* in the time step t. The second element denotes an increase of $s_{i}^{R}\left(t\right)$ caused by a change in the weight of the node *j* that is a neighbor of node *i*.

D.ii. The addition of *n*_4_ new edges between the object and intermediary.

(A26) $$\frac{ds_{j}^{R}}{dt}=p_{5}n_{5}m\left(\mu_{0}+\delta \right)\Pi \left(s_{j}^{R}\right)+p_{5}n_{5}m\underset{j\in \Upsilon \left(i\right)}{\sum }\delta \frac{\mu_{ij}\left(t\right)}{s_{i}^{R}\left(t\right)}\Pi \left(s_{i}^{V}\right)$$

On the right side of the Equation (A25), the first element represents an increase of $s_{i}^{R}\left(t\right)$, in which the change in the weight of the edge is caused only by the node *i* in the time step t. The second element denotes an increase of $s_{i}^{R}\left(t\right)$ caused by a change in the weight of the node *j* that is a neighbor of node *i* (Figure 4b).

E. The rejection of an intermediary service by a certain object

(A27) $$\frac{ds_{i}^{R}\left(t\right)}{dt}=-p_{7}n_{7}\left(\mu_{0}+\delta_{3}\right)-\underset{j\in \Upsilon \left(i\right)}{\sum }\delta \frac{\mu_{ij}\left(t\right)}{s_{i}^{R}\left(t\right)}\Pi \left(s_{i}^{V}\right)$$

F. Decreasing of the strength of an intermediary

(A28) $$\frac{ds_{i}^{R}\left(t\right)}{dt}=-p_{8}\gamma_{2}s_{i}^{R}\left(t\right)Ρ\left(s_{j}^{R}\right)$$

To solve Equations (A22)–(A28), we use the same procedures as for solving equations of objects (see (A7) to (A18)). We get similar equations:

(A29) $$s_{j}^{R}\left(t\right)\approx n_{2}\mu_{0}{\left(\frac{t}{t_{j}}\right)}^{\Gamma_{3}^{’}}$$

where

(A30) $$\Gamma_{3}^{’}=\frac{p_{1}n_{1}(\varepsilon_{0}+\delta_{1})+p_{2}n_{2}\left(\mu_{0}+\delta_{2}\right)+p_{3}n_{3}\mu_{0}+p_{4}n_{4}(\varepsilon_{0}+2\delta_{4})+p_{5}n_{5}m(\mu_{0}+2\delta )-p_{7}n_{7}\left(\mu_{0}+2\delta_{3}\right)}{\Gamma_{1}^{’}}-\frac{p_{8}\gamma_{2}}{p_{3}}$$

and

(A31) $$\Gamma_{1}^{’}=\frac{p_{1}n_{1}(\varepsilon_{0}+\delta_{1})+p_{2}n_{2}\left(\mu_{0}+\delta_{2}\right)+p_{3}n_{3}\mu_{0}+p_{4}n_{4}\left(\varepsilon_{0}+2\delta_{4}\right)+p_{5}n_{5}m\left(\mu_{0}+2\delta \right)-p_{7}n_{7}\left(\mu_{0}+\delta_{3}\right)}{1+p_{8}\gamma_{2}}$$

where $p_{8}\gamma_{2}>0$ and $\Gamma_{1}^{’}>0$.

We get for distribution $s_{j}^{R}$:

(A32) $$P\left(s^{R}\right)=H’{\left(s^{R}\right)}^{-\xi^{’}}$$

where

(A33) $$H^{\prime }=\frac{1}{\Gamma_{3}^{’}}{\left(n_{2}\mu_{0}\right)}^{\frac{1}{\Gamma_{3}^{’}}}$$

(A34) $$\xi^{\prime }=\frac{1}{\Gamma_{3}^{’}}+1$$

## Appendix C. Data for the Model Validation

Our goal was to perform simulations [61] with data that would approach some real tripartite network. Although tripartite networks can be found in social, biology, and information as well as in other areas, the only available way for us to obtain approximate parameter values for our model validation was to conduct a study on the tripartite network that appears in e-commerce. This tripartite network consists of users (users), price comparison sites (intermediaries), and e-shops (objects). Participants of the study were students. They were to write down when they used a price comparison site, or when they went directly to some e-shops. They were supposed to do it for more than two months. To find out the data concerning e-shops, we also addressed 32 e-shops. The questions we asked were about the number of user accesses, directly or through the price comparison websites, and how a price comparison site affects their revenue and more.

We processed the acquired data, and estimated values of the parameters of our model (Table A1). For example, one of the conclusions stated that almost 50% of respondents regularly used price comparison websites for their purchases, and 33% used them irregularly. Altogether 83% of respondents used price comparison websites because it helped them to navigate in the extensive offers of e-shops. From these data, we have estimated *p*_4_ and *p*_5_. We have proceeded similarly when estimating the values of other parameters. The *δ_i_* parameters mean how the respective actors on the site interact. For example, *δ_1_* means a parameter for local weight shift if a new e-shop is created. This will also affect existing e-shops. They must somehow react on a new competitor, for example, to adjust the price of the product, of the shipping or to respond in similar way. Based on this research and previous works [62,63,64], we estimated the parameters values as described in Table A1.

**Table A1:** List of the parameter’s values used for analytical calculations and simulations.

Parameter	Assigned Value
The probabilities of an entity formation	p_1_ = 0.020 the probability of new user formation p_2_ = 0.010 the probability of new object formation p_3_ = 0.001 the probability of a new intermediary formation p_4_ = 0.630 the probability of a creating a new edge between the existing user and an existing object p_5_ = 0.309 the probability that users will choose an intermediary which they use to go the object p_6_ = 0.005 the probability of decreasing the strength of an object p_7_ = 0 probability that the object will refuse to use the service of an intermediary p_8_ = 0 the probability of decreasing the strength of an intermediary
The number of newly generated edges	n_1_ = 2 new user generates n_1_ edges to intermediaries n_2_ = 2 new object generates n_2_ edges to intermediaries n_3_ = 5 new intermediary generates n_3_ edges from intermediary to object n_4_ = 1 n_4_ existing users will join existing objects n_5_ = 1 user choose n_5_ intermediariesm = number of objects that the user will contact after he the chooses them on this intermediary n_6_ = 2 the reduction in the strength of n_6_ objects n_7_ = 1 the number of edges removed after an object will not want further to use services of intermediaries n_8_ = 1 the reduction in the strength of n_8_ intermediaries
The weights of new edges	ω_0_ = 0.3 the weight of a new edge from user to object μ_0_ = 0.2 the weight of a new edge from intermediary to object ε_0_ = 0.3 the weight of a new edge strength from a user to an intermediary
Local shifts of weight of edges	δ_1_ = 0.0020 parameter of increasing of weight of relevant existing edges influenced by creation of a new edge from a new user to an intermediary δ_2_ = 0.0020 parameter of increasing of weight of relevant existing edges influenced by creation of a new edge from a new object to an intermediary δ_3_ = 0.0010 parameter of increasing of weight of relevant existing edges influenced by creation of a new edge from intermediary to object δ_4_ = 0.0010 parameter of increasing of weight of relevant existing edges influenced by creation of a new edge from a user to an intermediary δ = 0.0001 parameter of increasing of weight of relevant existing edges influenced by creation of a new edge from the intermediary to the object
Parameter of relevant node strength decreasing	γ_1_ = 0.01 parameter of object strength decreasing γ_2_ = 0.01 parameter of intermediary strength decreasing
Initial values for simulations	*U*_0_ = 50, *V*_0_ = 5, *E*_0_ = 10
